# Vonoprazan Therapy in Gastroesophageal Reflux Disease (GERD): Effects on the Severity and Frequency of GERD in Relation to Hiatus Hernia

**DOI:** 10.7759/cureus.97186

**Published:** 2025-11-18

**Authors:** Adnan Ur Rehman, Muhammad Shoaib Khan, Muhammad Iltaf, Wiqas Ahmad, Naeem Jan

**Affiliations:** 1 Gastroenterology and Hepatology, Hayatabad Medical Complex Peshawar, Peshawar, PAK

**Keywords:** gastroesophageal reflux disease (gerd), hiatus hernia, potassium-competitive acid blockers (p-cabs), proton pump inhibitors, vonoprazan

## Abstract

Objective

This study aimed to assess vonoprazan’s effectiveness in reducing the severity and frequency of gastroesophageal reflux disease (GERD) symptoms in relation to hiatus hernia.

Methods

This prospective single-arm cohort study (before and after treatment) was conducted at Hayatabad Medical Complex, Peshawar, from January 1, 2025, to August 31, 2025. It enrolled 110 patients with GERD, based on clinical evaluation and endoscopic confirmation to exclude other pathologies and evaluate for the presence of hiatus hernia. Inclusion criteria included patients aged 18-65 years presenting with typical GERD symptoms, such as heartburn or regurgitation. Patients were excluded if they had a history of gastrointestinal surgery, esophageal malignancy, pregnancy, lactation, and significant hepatic or renal impairment or were on concurrent acid-suppressive medications. All patients were given 20 mg vonoprazan monotherapy for four weeks. Symptom severity and frequency were assessed pre- and post-treatment using a validated structured questionnaire. Clinical response evaluation and subgroup analysis were performed to evaluate treatment outcomes in patients with and without hiatus hernia.

Results

The total number of patients was 110, with a mean age of 43.65 ± 9.90 years and a mean BMI of 26.81 ± 2.76. Before treatment, 33 (30.0%) had severe symptoms, and 58 (52.7%) reported symptoms more than four times per week. Following treatment, 97 (88.2%) reported complete symptom resolution, and symptom frequency decreased significantly. Among the 32 (29.1%) with hiatus hernia, 20 (62.5%) became symptom-free. Analysis showed a significant reduction in both symptom severity and frequency (p < 0.001).

Conclusion

Vonoprazan improved the severity and frequency of GERD symptoms significantly, with better outcomes in patients without a hiatus hernia. It offers an effective alternative in GERD management.

## Introduction

Gastroesophageal reflux disease (GERD) is a chronic and often progressive gastrointestinal disorder caused by the retrograde flow of gastric contents into the esophagus. Gastric acid reflux leads to various symptoms such as heartburn, regurgitation, and chest discomfort and, in severe cases, can result in mucosal damage manifesting as erosive esophagitis (EE) [1,2]. GERD significantly impairs patients’ quality of life, work productivity, and sleep, and it imposes a significant clinical and financial burden on healthcare systems globally [3].

Proton pump inhibitors (PPIs) have been the mainstay of GERD treatment for a long time. They suppress acid production by irreversibly inhibiting the H⁺/K⁺-ATPase pump in parietal cells. However, they have limitations, including a delayed onset of action and food-dependent activation, and variable patient responses have led to suboptimal symptom relief in a substantial subset of patients. It has been observed that nearly 30-40% of individuals on PPIs continue to experience reflux symptoms, while others relapse upon discontinuation, prompting the need for more effective alternatives.

Vonoprazan, a first-in-class potassium-competitive acid blocker (PCAB), represents a significant advancement in acid suppression therapy. Unlike PPIs, vonoprazan provides rapid, potent, and sustained inhibition of gastric acid secretion by competitively blocking the potassium-binding site of the proton pump, without requiring acid activation [4-6]. This pharmacological profile leads to more consistent 24-hour acid control and allows for more predictable clinical outcomes. Clinical trials and meta-analyses have demonstrated vonoprazan’s superior efficacy compared to traditional PPIs in healing EE, alleviating GERD symptoms, and preventing relapse [7-11].

Notably, vonoprazan has also proven effective in step-down and on-demand regimens, supporting its use in long-term GERD management strategies [12,13]. Its tolerability and favorable safety profile across diverse populations, including elderly patients and those with comorbidities, enhance its clinical utility [14].

In addition to pharmacological variables, anatomical abnormalities such as hiatus hernia have a known impact on the severity and recurrence of GERD. Hiatus hernia can exacerbate reflux symptoms by altering the angle of His and reducing lower esophageal sphincter pressure, thereby complicating treatment outcomes. Shinozaki et al. demonstrated that the presence of a hiatus hernia increases the likelihood of symptom recurrence after discontinuing vonoprazan therapy [15]. Similarly, Ochiai et al. reported that vonoprazan maintains efficacy even in patients with mild EE who exhibit structural predispositions such as small hernias [16].

Despite rising evidence supporting vonoprazan’s effectiveness, limited research has addressed its therapeutic performance specifically in patients with anatomical risk factors like hiatus hernia. As real-world outcomes may differ from controlled trial settings, this study aimed to evaluate the effectiveness of vonoprazan 20 mg once daily in reducing the severity and frequency of GERD symptoms over a four-week period and to compare treatment response between patients with and without hiatus hernia. This study aimed to guide more personalized and effective GERD management with vonoprazan by evaluating changes in symptom severity and frequency.

## Materials and methods

This prospective single-arm cohort study (before and after treatment) study conducted at a single center and was designed to evaluate the clinical efficacy of vonoprazan, a potassium-competitive acid blocker, in the treatment of GERD using a non-probability convenience sampling technique. It was conducted over a period of eight months from January 1, 2025, to August 31, 2025, at Hayatabad Medical Complex, Peshawar, following approval from the institutional ethics committee (approval no. 2102). Informed consent was obtained from all participants.

**Figure 1:**
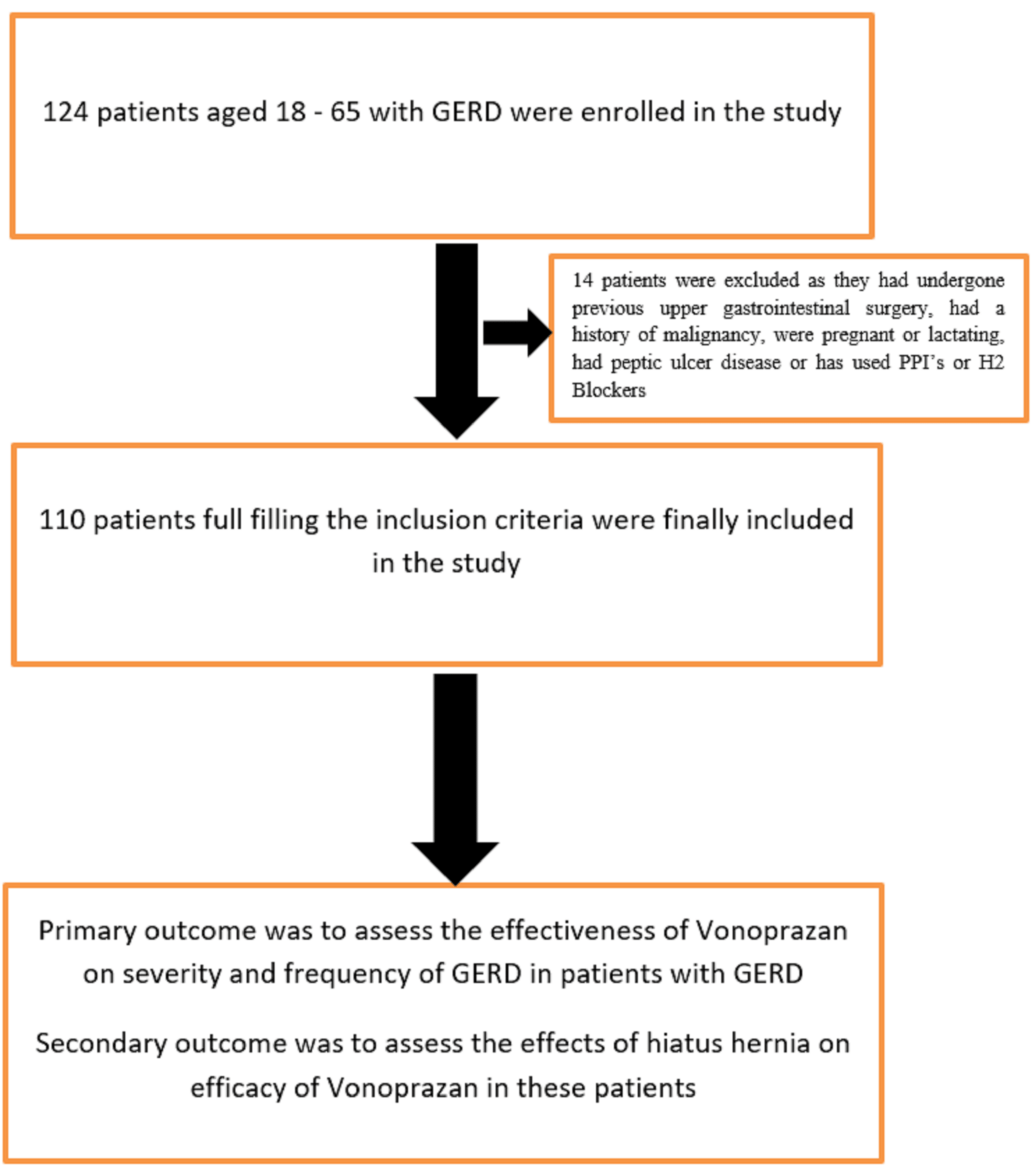
Study flow diagram

A total of 110 adult patients, aged 18 years and above, with GERD symptoms for at least three months, were included in the study. Upper GI endoscopy was done to exclude other pathologies and to determine the presence or absence of a hiatus hernia. Patients were excluded if they were pregnant, had a history of upper gastrointestinal surgery, Barrett’s esophagus, malignancy, or recent use of PPIs or H2 receptor antagonists, as shown in Figure 1.

All participants received vonoprazan 20 mg orally once daily for four weeks. Use of additional acid-suppressive agents was not permitted during the study period. Compliance was monitored through weekly follow-up phone calls and self-reported medication diaries.

For data collection, we developed our own questionnaire that included demographic information, relevant clinical details, and subjective scales assessing the severity and frequency of GERD symptoms. The questionnaire was reviewed and validated by three subject specialists and a member of the Institutional Review Board (IRB). A copy of the questionnaire is provided in the Appendix of this study. Demographic data included age, gender, and body mass index (BMI). At the same time, clinical parameters focused on the presence or absence of a hiatus hernia, a known anatomical risk factor influencing GERD severity and treatment outcomes. Symptom assessment involved categorizing GERD symptom severity subjectively as none, mild, moderate, or severe based on patient-reported discomfort levels and effects of GERD on daily routine. Symptom frequency was recorded as absent, less than two days per week, two to four days per week, more than four days per week, up to once daily, or more than once daily subjectively. These assessments were systematically conducted at two time points, i.e., prior to initiating therapy and after completing the four-week vonoprazan treatment. This methodology was essential for consistent data collection and allowed for the objective comparison of symptom progression, providing insights into the clinical efficacy of vonoprazan across patient subgroups.

Statistical analysis was performed using IBM SPSS Statistics for Windows, version 23.0 (released 2015, IBM Corp., Armonk, NY). Continuous variables were summarized as means and standard deviations, while categorical variables were expressed as frequencies and percentages. Patients were subcategorized post-treatment for analysis as no GERD and mild symptoms severity for analysis of severity and no symptoms, and two to four days per week symptoms for frequency analysis. Subgroup analyses were conducted to evaluate differences in response based on the hiatus hernia status. The McNemar test was used to calculate the p-value, and a p-value < 0.05 was considered statistically significant.

## Results

Table 1 demonstrates that the study comprised 110 patients with a mean age of 43.65 ± 9.90 years. The mean BMI was 26.81 ± 2.76 kg/m², with 63 (57.3%) having a BMI between 25 and 30. In terms of gender distribution, 64 (58.2%) were males and 46 (41.8%) were females. Regarding anatomical findings, 78 (70.9%) did not have a hiatus hernia, while 32 (29.1%) were found to have one, as shown in Table 1.

**Table 1:** Baseline characteristics and GERD symptom profile before and after treatment GERD: gastroesophageal reflux disease

Variable (n = 110)		Mean±SD n(%)
Age (years)		43.65 ± 9.90
Body mass index ( kg/m²)		26.81 ± 2.762
Body mass index groups	<25	34 (30.9%)
	25-30	63 (57.3%)
	31-35	13 (11.8%)
Gender	Male	64 (58.2%)
	Female	46 (41.8%)
GERD severity before treatment	Mild symptoms	15 (13.6%)
	Moderate symptoms	62 (56.4%)
	Severe symptoms	33 (30.0%)
GERD severity after treatment	No symptoms	97 (88.2%)
	Mild symptoms	13 (11.8%)
GERD frequency before treatment	2-4 days/week	41 (37.3%)
	> 4 days/week to once a day	58 (52.7%)
	More than once a day	11 (10.0%)
GERD frequency after treatment	Absent	97 (88.2%)
	<2 days/week	10 (9.1%)
	2-4 days/week	3 (2.7%)
Hiatus hernia	Not present	78 (70.9%)
	Present	32 (29.1%)

As shown in Table 1 and Table 2, the treatment led to a substantial improvement in both GERD severity and frequency among the total sample of 110 patients. Prior to treatment, none of the 110 patients were symptom-free. After treatment, 97 (88.2%) reported complete symptom resolution, while 13 (11.8%) had mild GERD symptoms (p < 0.001). Table 1 demonstrates that all cases of moderate (n = 62; 56.4%) and severe GERD symptoms (n = 33; 30.0%) improved with no patient having moderated to severe GERD after treatment. Regarding the frequency of GERD, 69 (62.7%) patients had GERD frequency ≥4 days per week. After treatment, GERD frequency significantly reduced in participants of the study, with 97 (88.2%) having no GERD while 13 (11.8%) having only two to four days per week symptoms (p < 0.001).

**Table 2:** Comparison of GERD severity and frequency before and after treatment according to the hiatus hernia status GERD: gastroesophageal reflux disease The McNemar test was used to calculate the p-value.

Variable (n = 110)			Before treatment n(%)	After treatment n(%)	p-value
GERD severity	With hiatus hernia (n = 32)	No symptoms	0 (0.0%)	20 (62.5%)	<0.001
		Mild symptoms	2 (6.3%)	12 (37.5%)	
		Moderate symptoms	8 (25.0%)	0 (0.0%)	
		Severe symptoms	22 (68.8%)	0 (0.0%)	
	Without hiatus hernia (n = 78)	No symptoms	0 (0.0%)	77 (98.7%)	
		Mild symptoms	13 (16.7%)	1 (1.3%)	
		Moderate symptoms	54 (69.2%)	0 (0.0%)	
		Severe symptoms	11 (14.1%)	0 (0.0%)	
GERD Frequency	With hiatus hernia (n = 32)	Absent	0 (0.0%)	20 (62.5%)	<0.001
		<2 days/week	0 (0.0%)	9 (28.1%)	
		2-4 days/week	6 (18.8%)	3 (9.4%)	
		> 4 days/week to once a day	15 (46.9%)	0 (0.0%)	
		More than once a day	11 (34.4%)	0 (0.0%)	
	Without hiatus hernia (n = 78)	Absent	0 (0.0%)	77 (98.7%)	
		<2 days/week	0 (0.0%)	1 (1.3%)	
		2-4 days/week	35 (44.9%)	0 (0.0%)	
		> 4 days/week to once a day	43 (55.1%)	0 (0.0%)	
		More than once a day	0 (0.0%)	0 (0.0%)	
Overall GERD	Severity (n = 110)	No symptoms	0 (0.0%)	97 (88.2%)	<0.001
		Mild symptoms	15 (13.6%)	13 (11.8%)	
		Moderate symptoms	62 (56.4%)	0 (0.0%)	
		Severe symptoms	33 (30.0%)	0 (0.0%)	
	Frequency (n = 110)	Absent	0 (0.0%)	97 (88.2%)	
		<2 days/week	41 (37.3%)	10 (9.1%)	
		2-4 days/week	58 (52.7%)	3 (2.7%)	
		>4 days/week to once a day	11 (10.0%)	0 (0.0%)	

Table 2 presents a comparison of GERD severity and symptom frequency before and after treatment, stratified by the presence or absence of hiatus hernia, along with overall outcomes for all participants (n = 110).

Among 32 patients with hiatus hernia, moderate to severe symptoms were initially present in 93.8% (n = 30) before treatment with vonoprazan. These symptoms resolved completely, with 62.5% (n = 20) of patients having no GERD and 37.5% (n = 12) experiencing only mild GERD symptoms after treatment. However, among 78 patients without a hiatus hernia, none were symptom-free before treatment, but 77 (98.7%) reported complete relief afterward. Among all these patients, mild symptoms decreased dramatically from 13 (16.7%) to just one (1.3%), while moderate and severe symptoms were fully resolved post-treatment. This difference in the reduction of GERD severity after treatment between patients with and without hiatus hernia is statistically significant (p < 0.001).

In terms of GERD symptom frequency, none of the patients with a hiatus hernia were asymptomatic before treatment. Post-treatment, 20 (62.5%) became symptom-free, nine (28.1%) reported symptoms less than twice a week, and only three (9.4%) reported symptoms two to four times per week. Notably, frequencies of more than four times per week and more than once daily, initially reported by 15 (46.9%) and 11 (34.4%) patients, respectively, were completely eliminated. Among those without a hiatus hernia, 77 (98.7%) became symptom-free post-treatment, while only one (1.3%) reported symptoms less than twice per week. Among these patients without hiatus hernia, 35 (44.9%) had symptoms two to four times per week, and 43 (55.1%) experienced symptoms more than four times weekly, both of which resolved entirely. This difference in reduction in GERD symptom frequency between patients with and without hiatus hernia is statistically significant (p < 0.001).

## Discussion

This prospective observational study demonstrated that vonoprazan therapy was associated with significant improvement in both the severity and frequency of GERD symptoms. Similar outcomes were observed in prior clinical trials comparing vonoprazan with high-dose PPIs, where comparable 24-hour heartburn-free rates were achieved with good tolerability [17]. In our study, 88.2% of patients were asymptomatic by the end of treatment duration, underscoring vonoprazan’s rapid and effective acid suppression.

One of the studies by Fass et.al. assessed the on-demand use of vonoprazan in patients with non-erosive reflux disease (NERD) and reported significant efficacy in managing intermittent reflux symptoms such as heartburn and regurgitation. This trial emphasized the benefit of vonoprazan in providing rapid symptom relief with flexible dosing [18]. While our study used a scheduled daily regimen, similar improvements in symptom resolution, particularly in mild cases, were observed, aligning with the findings from this flexible treatment approach.

In a study by Shinozaki et al., hiatus hernia was identified as a significant risk factor for symptom recurrence following the discontinuation of vonoprazan therapy, particularly in patients with moderate-to-severe reflux esophagitis. The authors emphasized that anatomical factors such as a compromised lower esophageal sphincter and diaphragmatic dysfunction may undermine the sustained effects of acid suppression [15]. In our study, patients with and without hiatus hernia showed statistically significant differences in improvement in GERD severity and frequency, as shown in Table 2. Moderate to severe GERD symptoms and frequency of GERD episodes ≥4 days/week completely resolved in the patient group not having a hiatus hernia, showing a significant response to vonoprazan in this group of patients, as shown in Table 2. These findings are consistent with the above-mentioned study results. However, in our study, patients were not followed after the study ended for any recurrence of symptoms, as described in the above-mentioned study.

The guideline by the American Gastroenterological Association (AGA) emphasizes that for GERD patients with large or para-oesophageal hiatus hernias and persistent symptoms despite medical therapy, surgical repair, such as fundoplication, is often necessary to restore anatomical integrity and optimize symptom control. This recommendation is based on the recognition that anatomical defects can accentuate reflux irrespective of acid suppression [19]. Our study results also demonstrated that patients with hiatus hernia had statistically significantly less improvement in symptoms compared to those with no hiatus hernia, as shown in Table 2, which is in agreement with the AGA guidelines statement.

A comprehensive review by Padwal et al. has emphasized vonoprazan’s strong therapeutic profile, citing its rapid onset, consistent acid suppression, and favorable tolerability across various GERD phenotypes, including erosive esophagitis, non-erosive reflux disease, and PPI-refractory cases. These analyses also noted low rates of adverse effects and high patient satisfaction [20]. Consistent with these observations, 88.2% of our participants reported a complete absence of symptoms after treatment, particularly in subgroups with moderate to severe symptoms and no hiatus hernia, as shown in Tables 1-2. This supports vonoprazan’s broad clinical applicability and reinforces its role as a practical option in GERD management.

One of the studies by Howden et al. conducted a comparative analysis between vonoprazan and conventional PPIs, demonstrating that vonoprazan provided more rapid, potent, and consistent acid suppression, resulting in superior symptom relief, particularly in patients with more severe forms of GERD. The authors noted enhanced mucosal healing rates and improved patient-reported outcomes with Vonoprazan [21]. Consistent with these findings, our data confirmed reliable symptom control in patients with moderate-to-severe GERD. Overall, 88.2% of all patients showed complete symptom resolution with vonoprazan therapy at the end of the study duration, as depicted in Table 1, further supporting the clinical advantage of vonoprazan over traditional PPI therapy.

A study by Simadibrata et al. assessed the efficacy of vonoprazan in patients with refractory GERD and found that vonoprazan demonstrated superior symptom relief and mucosal healing over other comparators. The authors highlighted vonoprazan’s rapid onset of action and consistent acid suppression as key advantages in managing difficult-to-treat cases [22]. Although refractory GERD patients were excluded, the significant symptom improvement observed in our cohort reflects similar outcomes, reinforcing vonoprazan’s effectiveness as a first-line treatment option. In our study, 86.4% patients had moderate to severe symptoms, and all showed excellent response to vonoprazan therapy with complete resolution of symptoms at the end of the study (p < 0.001), as shown in Tables 1-2.

A recent clinical update by the American Gastroenterological Association (AGA) emphasized the potential role of PCABs, such as vonoprazan, as a preferred option for patients requiring rapid and consistent acid suppression. The update highlighted vonoprazan’s superior pharmacological profile, including its faster onset and sustained acid control compared to PPIs, particularly beneficial in patients with severe or high-frequency symptoms [23]. Our study aligns with this recommendation, as patients with frequent GERD episodes or moderate to severe symptoms experienced significant (p<0.001) symptom resolution following vonoprazan therapy, shown in Table 2. Our study showed that 86.4% patients having moderate to severe symptoms had a complete response to vonoprazan therapy, whereas out of 62.7% of patients having GERD frequency of two to four days /week or more, only 2.7% had mild symptoms at the end of the study, as shown in Table 2, thus further reinforcing the value of early PCAB integration in GERD management.

In a study conducted by Xiao et al. on Chinese patients, the effectiveness of vonoprazan in routine GERD management was assessed. They reported high rates of patient satisfaction, rapid symptom relief, and strong adherence to therapy, even outside controlled trial environments. The study also highlighted vonoprazan’s consistent efficacy across different demographic subgroups, including those with anatomical risk factors [24]. Similarly, our findings reinforce these outcomes, as 93.8% of those patients with hiatus hernia having moderate to severe symptoms experienced notable symptom improvement and complete resolution of symptoms, as shown in Table 2, underscoring vonoprazan’s practicality in diverse real-world scenarios.

This study has several limitations, including its single-center design, limited sample size, short follow-up duration, and absence of post-treatment endoscopic evaluation or pH monitoring. In addition, the exclusion of refractory cases and reliance on self-reported symptom scores may limit the generalizability of the findings. Despite these constraints, the study provides valuable real-world evidence on vonoprazan’s efficacy in GERD management. It demonstrated significant improvement in GERD severity and frequency, even among patients with anatomical risk factors such as hiatus hernia. These results support vonoprazan’s integration into routine clinical practice as a potent alternative to traditional PPIs, especially in patients with moderate-to-severe disease. Future large-scale, multicenter studies with extended follow-up and objective endpoints are recommended to establish its long-term safety and effectiveness.

## Conclusions

Vonoprazan once daily significantly improved GERD symptoms severity and frequency with high rates of symptom resolution, specifically in the moderate-to-severe symptom group of patients and in those with more than four days per week frequency. Patients without a hiatus hernia responded more favorably with significant improvement in GERD severity, though those with a hernia also showed notable improvement, but not as significant as in those without a hiatus hernia. These results support vonoprazan as a safe and effective option for GERD management, especially in cases needing rapid and sustained relief and in those where an anatomical abnormality like a hiatus hernia is not present. As far as gender is concerned, it has no significant effect on the efficacy of vonoprazan in the management of GERD.

## Figures and Tables

**Figure 2:**
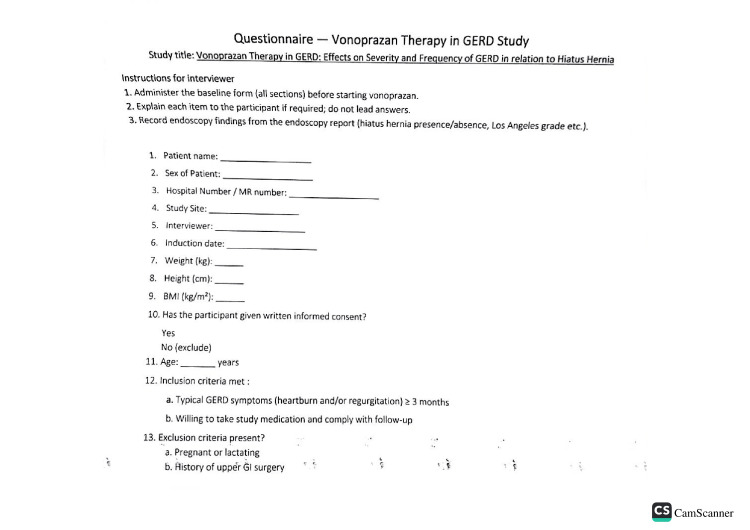
Questionnaire page 1

**Figure 3:**
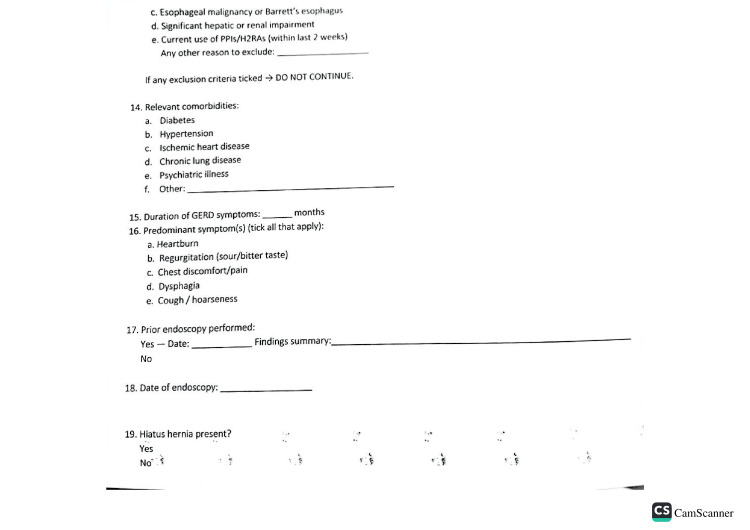
Questionnaire page 2

**Figure 4:**
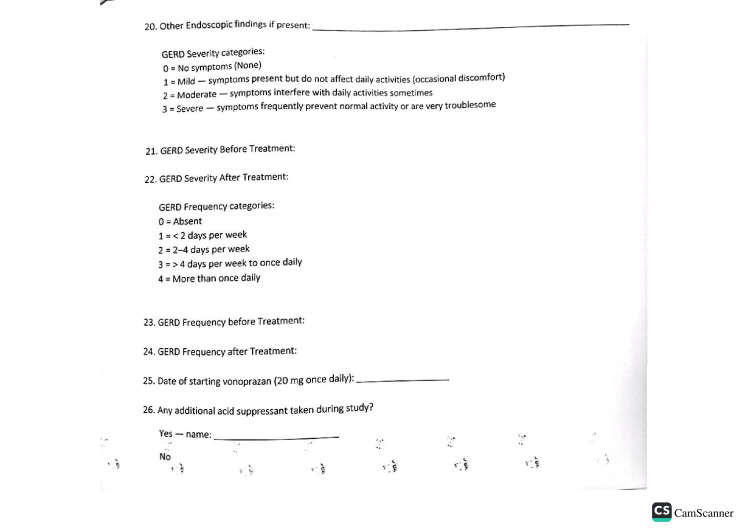
Questionnaire page 3

**Figure 5:**
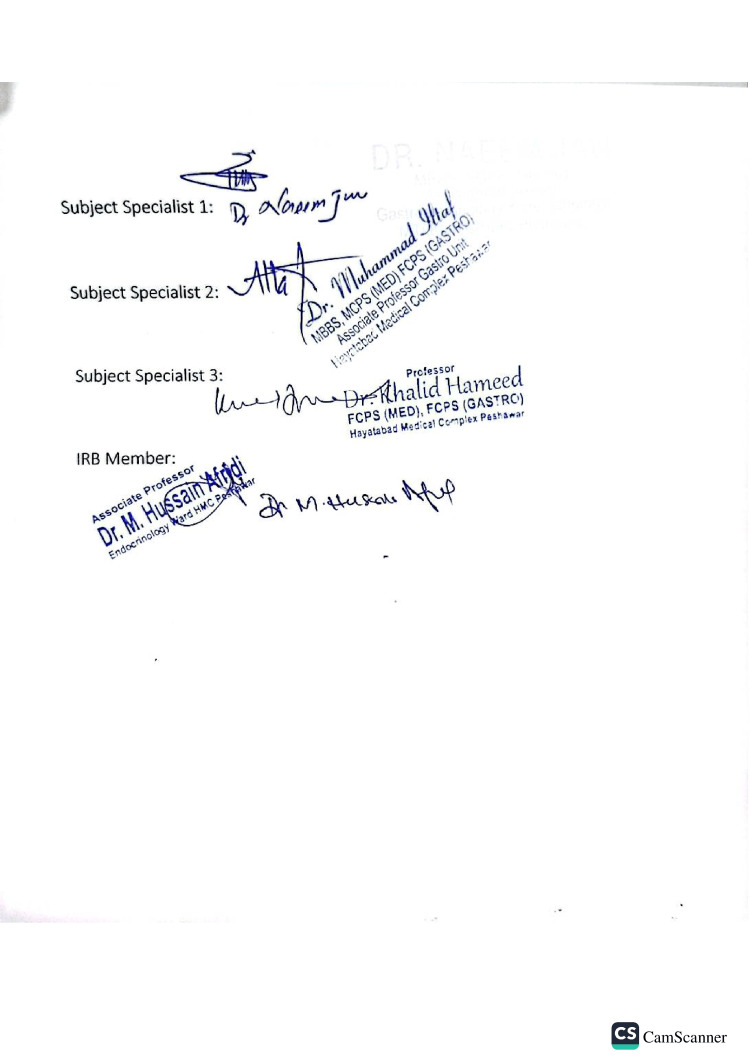
Questionnaire page 4

## Appendices

Study questionnaire (developed by the authors) 
